# Comparison of the efficiency of various muscle transposition procedures using a novel three-dimensional model

**DOI:** 10.1371/journal.pone.0204078

**Published:** 2018-09-18

**Authors:** Ju-Yeun Lee, Han Woong Lim, Jungmin Yoon, Jae Eung Oh, Kyung-Ah Park, Sei Yeul Oh

**Affiliations:** 1 Department of Ophthalmology, Samsung Medical Center, Sungkyunkwan University School of Medicine, Seoul, Republic of Korea; 2 Department of Ophthalmology, College of Medicine, Hanyang University, Seoul, Republic of Korea; 3 R&D Center, SEMES CO., LTD, Cheonan, Republic of Korea; 4 Division of Mechanical Engineering, Hanyang University School of Mechanical Engineering, Seoul, Republic of Korea; Faculty of Medicine, Cairo University, EGYPT

## Abstract

**Aim:**

To investigate the performance of a newly developed three-dimensional (3D) biomechanical model in various transposition procedures for correction of complete sixth nerve palsy with educational purpose.

**Methods:**

A 3D biomechanical eye model was created using Hyperworks software based on geometry data and the biochemical properties of the eyeball and extraocular muscles. A complete sixth nerve palsy model was achieved via modification of lateral rectus muscle strength. Four different muscle transposition procedures (the Hummelsheim, Jensen, Foster, and muscle union procedures) were set up, and the objective surgical effect of each procedure was calculated using 3D model simulation.

**Results:**

In the 3D simulation, sixth nerve palsy was modeled by rotating the eye 34.16 degrees in the medial direction, consistent with 70 prism diopter (PD) esotropia. In surgical model simulation, the Hummelsheim procedure resulted in a 28 PD reduction of total deviation, the Jensen procedure achieved a 34 PD reduction, the Foster procedure led to a 57 PD reduction, the muscle union procedure yielded a 57 PD reduction in esotropia in sixth nerve palsy.

**Conclusion:**

The 3D simulation provided a consistent model of sixth nerve palsy and objective data excluding the potential for variation of surgical skill. It could also help predict surgical outcomes.

## Introduction

In 1908, Hummelsheim reported a muscle transposition procedure for the treatment of lateral rectus muscle palsy [1]. Since then, a variety of transposition procedures have been used to address esotropia and the loss of abduction that results from complete sixth nerve palsy [2]. In 1964, Jensen reported a procedure wherein the lateral half of the superior or inferior rectus is joined to the lateral rectus [3]. Unlike Hummelsheim’s procedure, Jensen’s requires muscle union without tenotomy, therefore Jensen’s procedure is generally associated with reduced postoperative risk of anterior segment ischemia [3–5]. In 1997, Foster reported that vertical muscle transposition, augmented with lateral fixation sutures, increased abduction force while maintaining adduction in patients with Duane’s syndrome or sixth nerve palsy [6].

Although the Hummelsheim, Jensen and Foster methods are well established, a variety of modifications have been introduced [7–10]. Those modifications of the muscle transposition procedure aim to strengthen the abductive effect in order to correct complete sixth nerve palsy and to minimize complications such as anterior segment ischemia. Antje et al. reported a modified transposition procedure with comparable surgical results to the classic Hummelsheim method [11]. In recent years, Maria et al. compared the clinical outcomes of three different vertical rectus muscle transposition procedures including full-tendon transposition (FTT), FTT with 4-mm resection before reinsertion, and FTT with myopexy suture [12]. FTT with 4-mm resection before reinsertion corrected esotropia and improved abduction to the greatest degree.

Along with a number of transposition procedures, we introduced a muscle union procedure for paralytic strabismus with good postoperative results [10]. The procedure was effective in patients with a large angle of deviation, and no significant complications including anterior ischemia were reported.

Although there have been numerous studies on muscle transposition, most published articles only reported the clinical results of a single procedure, had a relatively small number of cases, and were based on the experience of the author using a given transposition procedure to treat patients affected by a variety of forms of strabismus, including unilateral and bilateral sixth nerve palsy as well as Duane syndrome and multiple cranial nerve palsies. Hence, we conducted this research as an objective comparison of the effect of various transposition surgeries.

For bio-simulation of the orbit and strabismus, computer modeling has been used [13,14]. Demer et al. predicted the effects of extraocular pulley and surgery in a strabismus model using the Orbit^TM^ program [14]. Subsequently, the SEE++ system, which is the realization of a biomechanical model of the human eye based on concepts originally invented in the Orbit^TM^ program, was also introduced [15].

Based on these computer programs, in the present study, we created a three-dimensional (3D) model eye to facilitate the conception, creation and visualization of muscle transposition surgery for complete sixth nerve palsy. Then, we evaluated surgical outcomes from different muscle transposition procedures (Hummelsheim, Jensen, Foster, and muscle union procedures). The purposes of this study was to investigate the performance of the newly developed 3D biomechanical model in various transposition procedures to correct complete sixth nerve palsy with educational purpose.

## Methods

**Fig 1:**
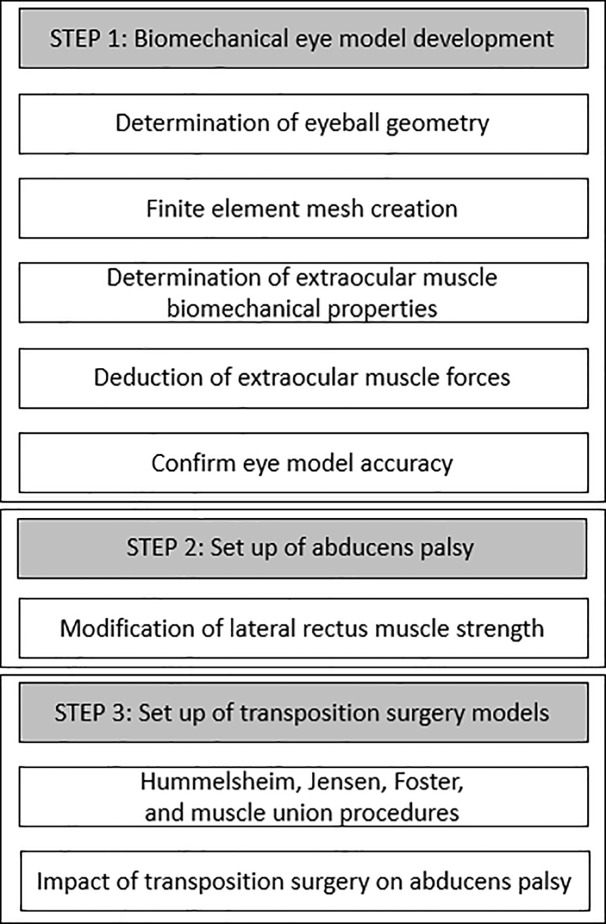
A process of simulation model setup.

Biomechanical modeling is a branch of mechanics that examines forces acting upon biological structures and the effects induced by these forces. Thus, it can simulate the physiologic functions of the human eye using parts, properties and parameters that are comparable. A diagram of the simulation model setup used in this study is summarized in Fig 1.

We used the finite-element method to analyze the dynamic effects of extraocular muscles on the rotation of eyeball, which accounts for the geometry of the modeled structure, the forces applied to that structure, and the elastic properties of the structure. All linear static finite-element analyses of the models were conducted using HyperWorks 11.0^®^ (Altair Engineering, Inc., USA).

In our study, geometry data were imported from the computer aided 3-D interactive application **(**CATIA) system, which was selected for this biomechanical project due to its extensive use in the biomechanical industry [16]. CATIA is a powerful software tool that consists of geometric 3D modeling, drafting and analysis capabilities that can be combined with the CATIA geometry interface and CATIA mathematical subroutines package to access and modify the database.

### Geometry and eyeball modeling

The assumptions for human eye simulation were as follows:

- The eyeball is completely spherical.
- The extraocular muscles are a one-dimensional element.
- The central axis of the eyeball is fixed.
- Only rotational motion is allowed along the x, y and z axis.
- Orbital connective tissue and oblique muscles are not considered.

**Fig 2:**
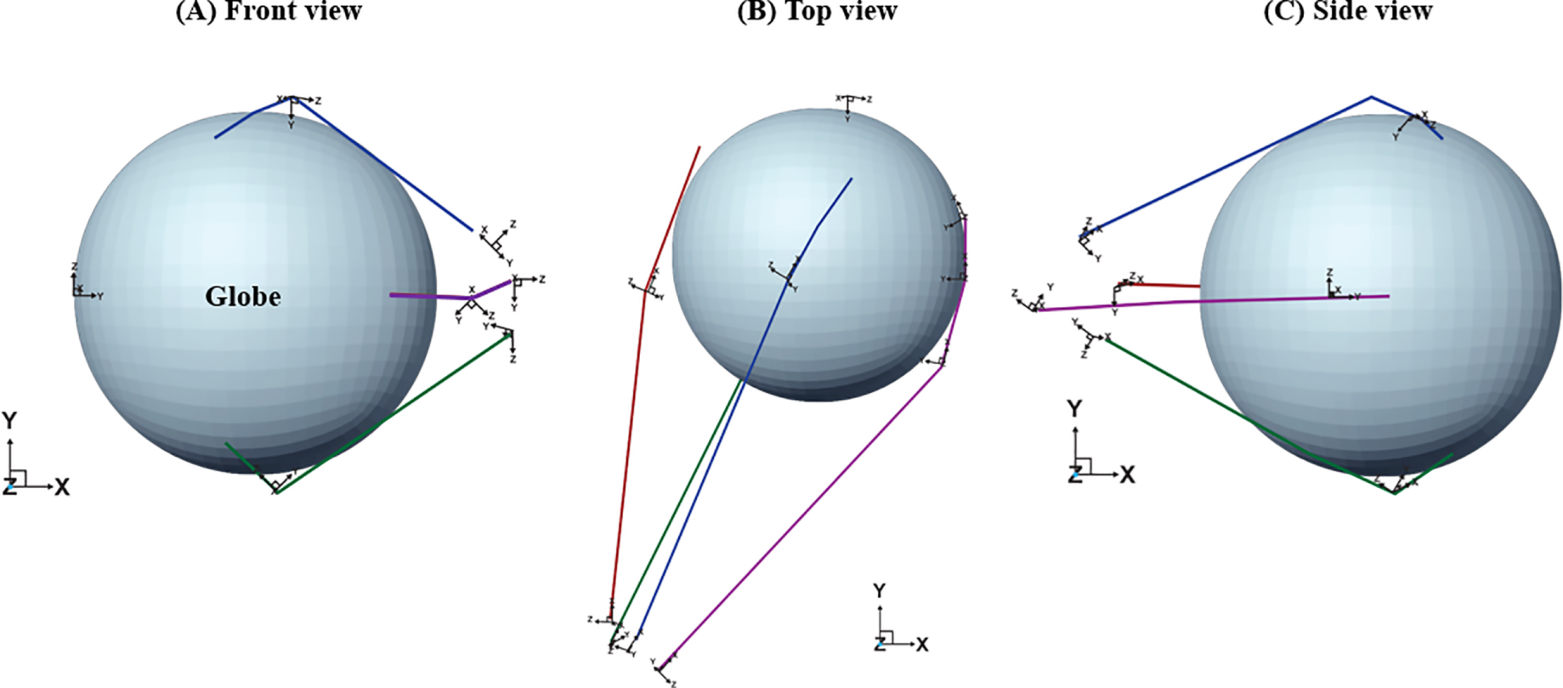
Construction of a 3D eye model with extraocular muscles and the pulley system. An eyeball was constructed as a homogenous sphere with a 12-mm radius. The rectus muscles were supposed to act as a spring model, and the origin of the rectus muscle was fixed at a single point. A pulley system was incorporated into the rectus muscles based on previous geographic data [18].

**Fig 3:**
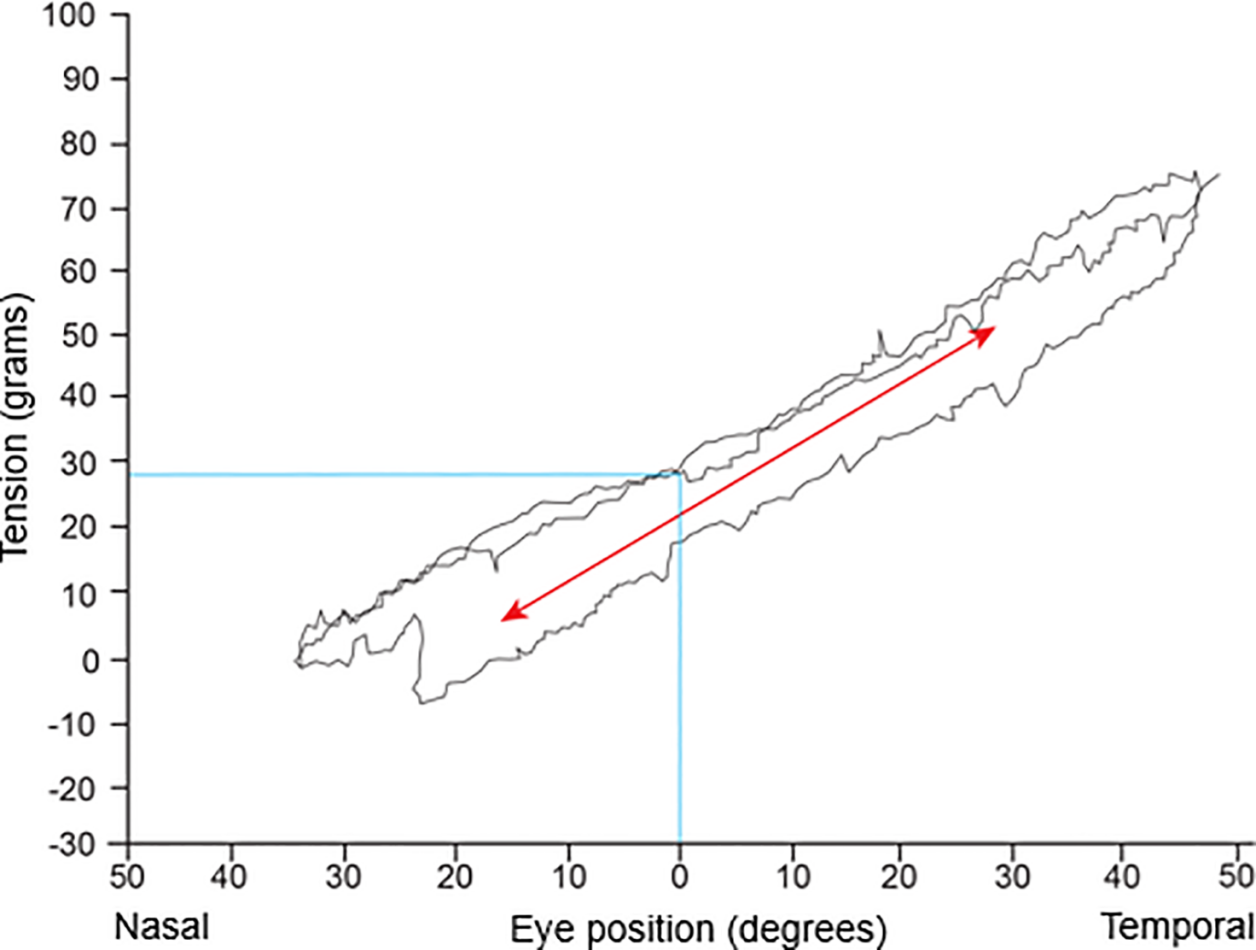
Relationship between tension record and temporal rotations of a normal eyeball. The angle of orbital rotation according to force within the scope of eyeball rotation established the linearity [28]. Based on the result, we assumed that the extraocular muscle acts as a spring.

Orbit^TM^ biosimulation 1.8 program (Eidactics, San Francisco, Calif) and SEE++ software program (RISC software GmbH, USA) were employed to obtain geometric data for eyeball modeling. Based on geometric data [17–19], an eyeball was constructed as a homogenous sphere with a 12-mm radius (Fig 2). The rectus muscles were supposed as a spring model to attain the effect of the force of rectus muscles (Fig 3). The origin of the rectus muscle was fixed at a single point (similar to the annulus of Zinn), and liberal movement of the rectus muscle was allowed only within the muscle vector direction. Muscle parameters in this study were based on published data by Miller et al. [18] A pulley system was incorporated into the rectus muscles. The pulley was located the same distance from the insertion site of each rectus muscle.

### Material properties

The mechanical properties of the eyeball and rectus muscles have not been completely elucidated. For this simulation, all materials were assumed to be linearly elastic, isotropic, and incompressible. The modulus of elasticity and Poisson's ratio were used to determine the biomechanical behavior of the rectus muscles based on the results of a previously published study [20]. The modulus of elasticity was set as 4 x 10^9^ Pa in the eyeball and 7.9 x 10^9^ Pa in the rectus muscle. Poisson's ratio was determined as 0.43 in the eyeball and 0.4 in the rectus muscle. The detailed biomechanical properties of the eyeball and rectus muscle are presented in Table 1.

**Table 1:** Biomechanical properties of the model eyeball and rectus muscle.

	Biomechanical property	Value
**Eyeball**	Modulus of elasticity	4 x 10^9^ Pa
	Poisson's ratio	0.43
	Density	1200 kg/m^3^
**Rectus muscle**	Modulus of elasticity	7.9 x 10^9^ Pa
	Poisson's ratio	0.4
	Density	930 kg/m^3^
	Damping	0.06

### Expression of extraocular muscle forces

The bearing tension of each rectus muscle was analyzed to maintain the primary position of the eyeball using computer-aid engineering tools. In the primary position, the mean bearing tension of the medial rectus (MR) muscle was 25.6g. The relative bearing tension of the other rectus muscles was calculated based on the bearing tension of the MR muscle (Table 2). The calculated bearing tension of the lateral rectus (LR), superior rectus (SR) and inferior rectus (IR) muscles was used as a paralytic strabismus model, and did not vary during the study.

**Table 2:** Calculated bearing tension of the four rectus muscles. We first set the medial rectus muscle tension obtained from a previous study and evaluated the subsequent reaction forces of the other rectus muscles to maintain the standard eyeball position established based on geometric data. In the standard model, superior rectus and inferior rectus were more medially located, so the relative lateral rectus tension was estimated to be larger than medial rectus tension.

Muscle	Bearing tension (grams)
Medial rectus	25.6
Lateral rectus	30.3
Superior rectus	22.0
Inferior rectus	22.2

In order to demonstrate the reproducibility and accuracy of the simulated model, we applied various MR bearing tension values according to the amount of eyeball deviation: 0, 10, 20, 30 and 40 degrees. The consistency between the original eyeball deviation setting and the amount of exodeviation resulting from the application of MR bearing tension was analyzed. A difference between the two values <5% was considered “acceptable”.

### Complete sixth nerve palsy modeling

As a consequence of a lesion on the sixth nerve, the contractile strength of the LR muscle can be reduced and its elastic parts thus have to be modified. In the SEE++ software system, this force reduction is carried out in the muscle data dialog as the basis for the simulation. In the field, the total abduction vector of the LR muscle can be reduced by changing this value from 100 to 0% (Table 3). Based on the results of this modification, the eye model was rotated 34.16° in the medial direction. This setting was consistent with 70 prism diopter (PD) esotropia in clinical sixth nerve palsy.

**Table 3:** Degree of ocular deviation according to the abduction vector force of lateral rectus muscle.

Case (%)	Abduction vector force (gram)	Ocular deviation (degrees)
0	0	34.16
20	6.06	27.33
40	12.12	20.50
60	18.18	13.67
80	24.24	11.08

### Transposition surgery models

**Fig 4:**
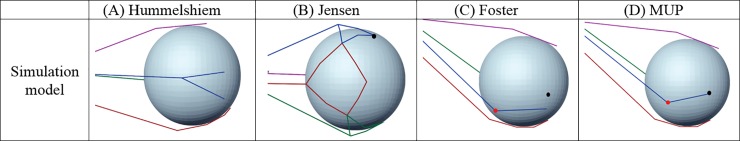
Simulation of the various muscle transposition procedure. (A) Hummelsheim, (B) Jensen, (C) Foster, (D) muscle union procedure (MUP) (blue line: superior rectus, green line: inferior rectus, red line: lateral rectus, purple line: medial rectus, red dot: pulley, black dot: original insertion of the superior rectus muscle). We changed muscle vectors, origins, and pulley locations consistent with each surgical method to realize the surgical effect and then evaluated temporal rotation degree of the eyeball using each biochemical model.

Hummelsheim, Jensen, Foster, and muscle union procedures were simulated. Each 3D model is presented in Fig 4. For surgical correction of simulated sixth palsy, strengthening of the paretic muscle is necessary. In setting up of Hummelsheim procedure model, the surgery was carried out by transposing half of the tendon of the SR and IR muscles to the LR muscle by 45 degrees. Afterwards, the modified strength of the LR muscle was calculated by Hook’s law as follows:

(ΔF=kx=k(M′−M) (1)

where F is tension, k is muscle force (muscle spring constant) which refers to the slope of the force according to the angle of eyeball rotation, x is change in muscle length, M’ is muscle length in the strabismus model, and M is muscle length in the normal model. M’ and M were obtained retrospectively from each eye model already set up with geometry data.

In the Jensen procedure model, SR and IR muscles were divided into two equal parts and united with the LR muscle. The pulley was not moved in this model. The modified strength of the LR muscle was calculated in the same way as it was in the Hummelsheim procedure model.

In the Foster procedure model, surgery involved full transposition of the SR and IR muscles toward the LR muscle by 45 degrees. Afterwards, the effect of full transposition and posterior fixation was demonstrated by the pulley movement toward the LR muscle by 45 degrees.

In the muscle union procedure, the union point between the SR, IR and LR muscles was modified based on the Jensen procedure. According to the location of muscle union, this model was conducted in two ways. The muscles were united at the mid-point between the vertical muscles and the LR muscle (±30 degrees from the x-axis). In these models, the pulley was supposed to move toward the LR muscle by 15 degrees.

#### Effect of transposition surgery on sixth nerve palsy

In order to analyze the impact of the four different transposition procedures on sixth nerve palsy, each surgical model was applied to the model of complete sixth palsy previously created in this study. The reduction in esodeviation and mean recovery rate were investigated.

## Results

In the 3D model simulation, a strabismic position (sixth nerve palsy) was formed by rotating the model eye 34.16 degrees in the medial direction. This setting was consistent with 70 PD esotropia in clinical sixth nerve palsy. We illustrated surgical techniques using the 3D model and the degree of lateral rotation from muscle transposition procedures was calculated. The results showed 13.4 degrees external rotation in the Hummelsheim procedure, 16.9 degrees in the Jensen procedure, 28.3 degrees in the Foster procedure, 16.8 degrees in the simple muscle union procedure and 28.4 degrees in the augmented muscle union procedure.

The Hummelsheim procedure achieved a 40.8% (28 PD) reduction in total deviation, the Jensen procedure resulted in a 49.4% (34 PD) reduction, the Foster procedure led to an 82.9% (57 PD) reduction, the muscle union procedure yielded an 82.9% (57 PD) reduction in esotropia in sixth nerve palsy.

## Discussion

Many trials have evaluated the surgical outcomes of various muscle transposition procedures. However, objectively comparing the surgical effect of various surgical methods is clinically difficult because the technique used and surgeon experience vary from one study to another. In addition, most published studies have small sample sizes, include various types of strabismus and are retrospective in nature. Therefore, we attempted to compare surgical outcomes objectively with a 3D biochemical model.

In the present study, we used a novel 3D model simulation approach. Given the data obtained regarding the anatomic position and path of the extraocular muscles for the initial setting of 3D model simulation, we created muscle force and operated 3D model with maximal virtual reality. Using this biomechanical model, we compared four different muscle transposition procedures and evaluated residual deviation in sixth nerve palsy. We found that the Foster procedure resulted in a greater reduction of esodeviation than the Hummelsheim and Jensen procedures. Some findings are similar to surgical outcomes in previous reports. According to previous studies, the mean ocular reduction to the primary position was about 52PD in the Hummelsheim procedure with medial rectus recession [21], 36PD in the Jensen procedure [3], 41PD in the Foster procedure [22,23], and 52PD in the muscle union procedure [10]. The discrepancy in the amount of reduction between simulation and the previous results of the Hummelsheim procedure may result from the augmented effect of medial rectus recession in published studies.

The variation in results between these transposition procedures could be explained by various factors. One possible explanation relates to differences in the amount of tonic abducting force increase and passive elastic force increase via lengthening of the path of the vertical muscles [24]. Most muscle transposition procedures produce tonic abducting force by changing the force vectors of transposed muscles [6]. In a number of studies, the authors added posterior fixation of the sclera with muscle transposition to increase the tonic abducting force, and reported that this augmented procedure could produce a 50% increase in the tonic abducting force compared with the classic full-tendon muscle transposition method [6,22]. The tonic force of transposed muscles has been reported to increase when the distance between the transposed muscles and the affected LR muscle are reduced [6], but this varies between procedures. This phenomenon could also have affected the outcomes of this study.

Another plausible explanation of different results between these transposition procedures is a difference in muscle pulley movement that can produce a powerful abducting force. Muscle pulleys of the vertical rectus muscles are located 10.65mm superior to the globe center and 12.33mm inferior to the globe center [25]. During transposition procedures, the muscle pulleys of the vertical rectus muscles are pulled along the LR muscle, and get closer to the pulley of the LR muscle. Then, the vertical force of the vertical rectus muscle pulley action can enhance the horizontal force via parallel arrangement with the contractile force of the LR muscle [26]; this strengthens the abducting force, although the contractile force of the LR muscle is zero because the horizontal force of the vertical rectus muscle is consistent. Therefore, fine differences in these mechanical factors between procedures may lead to different outcomes of various transposition procedures.

We found that the muscle union procedure previously introduced had comparable outcomes for correcting esotropia in sixth nerve palsy compared with other muscle transposition procedures. Considering that the temporal half of the vertical rectus muscles (SR and IR) is connected to the LR muscle at a 10-mm posterior point to the LR insertion site using 5–0 non-absorbable suture in the muscle union procedure [27], it could produce compatible results with posterior fixation suture in augmented muscle transposition by changing muscle vectors in the same direction. In the muscle union procedure, we assembled the halves of the muscles altogether, then maximized the tonic abduction force by minimizing the gap between the transposed muscles and the affected LR muscle. In addition, by connecting the vertical rectus muscles and the LR muscle 10mm posterior to the LR muscle insertion, vertical rectus muscle pulley movement along the LR muscle could create powerful horizontal force [26].

It is of note that the values obtained by virtual simulation are objective. Although these values may vary from those found in clinical practice in that our biomechanical model was simulated with certain assumptions and simplifications, we tried to maximally specify the known mechanisms of muscle transposition surgery and realize them. Thus, we could elicit objective results based on clear concepts, and the results enable comparison of the effects of various muscle transposition procedures for use in surgical planning.

This study has some limitations. The 3D model used in this study represented pure sixth nerve palsy. It excluded the effects of connective tissue and oblique muscles and could not consider restriction of the MR muscle. Particularly, extraocular muscles were assigned unidirectional properties; thus, the results could differ from clinical practice. However, we set the 3D model by focusing on LR muscle paralysis, which largely contributes to ocular deviation in sixth nerve palsy, then we used the 3D model in virtual reality based on previously obtained data. Therefore, it is meaningful for comparing surgical outcomes between muscle transposition procedures in sixth nerve palsy using the 3D model.

In conclusion, we created a 3D model that predicts surgical outcomes and can contribute to surgical treatment planning for correction of sixth nerve palsy. We could also provide a baseline result for an improved 3D model of various classes of strabismus and surgery which is needed to be used for accurate predictions in establishing therapeutic plan. This 3D model may provide a consistent model of sixth nerve palsy and, through objective data, exclude possible variation in surgical skill and variation in the degree of ocular deviation. It can be also valuable for assessing other strabismus surgery, in terms of mechanism research, prediction of treatment outcomes, ophthalmic education and residents training. Well-controlled studies are warranted to compare surgical outcomes between these muscle transposition procedures to verify our results.
